# DNA Injury and Repair Systems

**DOI:** 10.3390/ijms19071902

**Published:** 2018-06-28

**Authors:** Guillermo T. Sáez

**Affiliations:** Department of Biochemistry and Molecular Biology, Faculty of Medicine and Odontology, Instituto de Investigación Sanitaria, Hospital Clínico de Valencia (INCLIVA), Service of Clinical Analysis, University Hospital Dr. Peset-FISABIO, University of Valencia, Avda, Blasco Ibañez 15, 46010 Valencia, Spain; Guillermo.saez@uv.es; Tel.: +34-96-3864160

The importance of preserving the integrity and sequence of DNA for the life of a species finds its highest expression in the development and evolutionary perfection of its DNA damage response (DDR). DDR must be preserved over the lifespan of an organism to prevent diseases. However, the function and regulatory mechanisms of DDR may be impaired in certain situations or diminished by aging, underlining the pathological processes of different diseases, in particular, cancers [1]. This has generated an immense amount of interest in acquiring more knowledge of this topic and conceiving new and promising experimental designs and lines of scientific work worldwide. An example of the growing interest in this area of biomedical research is the continuously accumulating data that has been collected through different webs of knowledge and scientific journals. Some of these new insights have been reported by expert scientific groups in this field in the last issue on the injury of genetic material and repair systems, the most relevant results of which are summarized below. In this issue, along with the corresponding scientific papers and reviews, different aspects of this subject have been approached from the biochemical and molecular point of view to examine their translational application.

Enzyme-induced somatic hypermutation (SHM) and immunoglobulin class switch recombination (CSR) are necessary processes for antigen affinity and for shaping the effector function of the humoral immune response, respectively. In relation to this process, recent research has provided more mechanistic insight into the regulation of the mutagenic enzymes RAG1/2 and activation-induced cytidine deaminase (AID) enzyme expression and activity in lymphocytes. The complex interplay between these mutagenic enzymes and the base excision and mismatch repair pathways has been reviewed in the context of genome stability [2].

Three reports have focused on specific proteins of the cell replication process and their implications regarding DNA damage and repair mechanisms: the p53 binding protein 1 (53BP1), the topoisomerase reverse gyrase 1 (TopR1), and the DNA replication helicase/nuclease 2 (DNA2) [3,4,5].

Due to its homeostasis importance, p53 is a matter of continuous interest. Here, 53BP1 was reviewed as an emergent and important key aspect of DNA damage response as a component of nuclear bodies (NBs). The regulation and dynamics of 53BP1 in nuclear bodies, its implications for the DNA damage process, and its relation to replication stress and associated degenerative diseases has been summarized and reviewed by Fernandez-Vidal et al. [3].

Reverse gyrases play an important role in DNA replication, recombination, and repair. One of its components, TopR1, introduces positive supercoils to circular DNA and is implicated in the maintenance of genome stability. The function of TopR1 in protecting against DNA damage in thermophile strain *Sulfolobus islandicus* has been demonstrated by enzyme inhibition using a CRISPR-mediated mRNA interference approach (CRISPRi). The authors of [4] concluded that TopR1 probably facilitates genome integrity maintenance by protecting DNA breaks against thermodegradation.

The DNA replication helicase/nuclease 2 (DNA2) protein is recognized to also play an important role in DNA replication, both in the nucleus and mitochondria, by acting as a helicase and nuclease. DNA2 interacts with over 300 genes, achieving important functions for many fundamental processes, including DNA replication, chromatin modification, and DNA repair. In this issue on DNA injury and repair, a complete review of DNA2 protein is reported showing its relevant roles and biological features, including telomere maintenance and cell cycle regulation. In their review, Pawlowska et al. addressed the question of the repair role and amplitude of this replication protein as well as its potential as a clinical marker and target molecule for cancer treatment [5].

The role of biochemical substrates from endogenous (acetaldehyde) and exogenous (aristolochic acid) origin in the induction of DNA damage and/or modifications have been analyzed by two research groups.

Acetaldehyde, a product of alcohol metabolism by aldehyde dehydrogenase 2 (ALDH2), has been presented as a highly reactive compound that causes various forms of genomic damage, including the formation of different DNA adducts, which in turn are associated with the induction of mutations and/or impaired DNA metabolism. This has led to its consideration as a definite carcinogen for the esophagus and/or head and neck. Recent advances from studies of acetaldehyde-mediated DNA adducts formation were reviewed by the group of Manabu Muto, who emphasized the role of DNA repair pathways, such as the Fanconi anemia (FA) pathway, as a prevention mechanism of carcinogenesis in the squamous epithelium of the upper aerodigestive tract [6].

In addition to acetaldehyde, other naturally occurring substrates with the ability of DNA adduct formation, such as the plant alkaloid aristolochic acid (AA), have been presented and their biological effects analyzed. The AA-DNA adducts have been proposed as biomarkers for the assessment of AA exposure and markers of AA-induced urothelial cancer as well as a way to evaluate the mechanism of its enzymatic activation and detoxification [7].

FA is a DNA-damage-related disease with a high tumor incidence. The role of specific related proteins in the well-known FA signaling pathway deserves special attention. Recent studies have provided useful information about the FA group D2 protein (FANCD2) in DNA damage response and principal features have been updated in this review. FANCD2 has been proposed as a veteran checkpoint-player coupling with a variety of cellular processes outside the FA signaling pathway by interacting with FA and non-FA protein partners for DNA damage repair and genome stability [8].

In close relation with the mechanism of carcinogenic susceptibility, the role of the protein PALB2 (partner and localizer of breast cancer 2 (BRCA2)) has been approached. This protein represents a critical factor for DNA repair by orchestrating the functions of other different repair proteins and therefore contributing to the preservation of DNA integrity. Mutations of PALB2 in the predisposition to breast cancer emphasize its role as a tumor suppressor protein [9].

In relation to methodological aspects, the paper by Schulz et al. describes a modified combined in vivo method to measure the time-course of DNA damage response in tumors using a canine model. The authors were able to follow dynamics of the comet tail intensity and H2AX foci during a course of radiation using a minimally invasive approach. They demonstrated that by integrating the resulting data into a dynamic mathematical method, DNA repair can be quantitatively investigated as time-courses of individual patients [10].

As has been known, DNA aberrations might affect the differentiation capacity of embryonic stem cells and increase their tumorigenicity. The importance of DDR during the cellular reprogramming of embryonic stem cells (ESCs) and its implication in tumorigenicity has been addressed in the review by Turineto et al. The presented data in this report highlight the importance of understanding the molecular mechanisms underlying genomic instability during cell reprogramming, a process that involves not only DNA damage response activation but also an additional selective reprogramming inhibition when genetic anomalies occur. For this purpose, different ways and mechanisms of DDR have been created to avoid DNA aberrations and to discriminate adverse from silent abnormalities during cell reprogramming [11].

In the article by Yang et al., the role of APE1 in neurodegenerative diseases and its relationship with the neuroprotective mechanisms of the activated GLP-1 receptor (GLP-1R) was reviewed. In this article, new and useful insights into the DNA repair system for studying potential treatments in neurodegenerative diseases were provided by the authors and further discussed [12].

Genotoxic treatments elicit DDR not only in cells that are directly exposed but also in cells that are not exposed to genotoxic reagents (bystander cells), a phenomenon commonly referred to as the bystander effect (BE). However, mechanisms underlying the BE remain elusive. In the present issue, it was reported that etoposide and ultraviolet (UV) exposure stimulate the production of microvesicles (MVs) in DU145 prostate cancer cells. MVs isolated from UV-treated DU145 and A431 epidermoid carcinoma cells as well as etoposide-treated DU145 cells induced phosphorylation of ataxia-telangiectasia mutated (ATM) at serine 1981 (indicative of ATM activation) and phosphorylation of histone H2AX at serine 139 (H2AX) in naïve DU145 cells. Annexin V, which neutralizes the formation of MVs derived from UV-treated cells, significantly reduced the MV-associated BE activities. The presented results provide evidence supporting that MVs are a source of the DNA damage-induced bystander effect [13].

Finally, a complete update on the role of chromatin remodeling to ensure the exposure of DNA lesion sites to DNA repair proteins in eukaryotes was presented with interesting features and biological characteristics. In this review, Stadler and Richie focused on the question of how the cellular DNA repair pathways overcome the chromatin barrier, how the chromatin environment is rearranged to facilitate efficient DNA repair, what the proteins are that mediate this reorganization process, and how the altered molecular architecture of chromatin is involved in the regulation of DNA damage responses [14].
